# Ingesting Self-Grown Produce and Seropositivity for Hepatitis E in the United States

**DOI:** 10.1155/2018/7980413

**Published:** 2018-07-15

**Authors:** Thomas M. Diehl, Daniel J. Adams, Cade M. Nylund

**Affiliations:** ^1^McGill University, Faculty of Medicine, Montreal, QC, Canada H3G 2M1; ^2^Department of Pediatrics, Naval Medical Center Portsmouth, Portsmouth, VA 23708, USA; ^3^Department of Pediatrics, F. Edward Hébert School of Medicine, Uniformed Services University of the Health Sciences, Bethesda, MD 20814, USA

## Abstract

**Background:**

Hepatitis E virus (HEV) is a major cause of hepatitis in developing and industrialized countries worldwide. The modes of HEV transmission in industrialized countries, including the United States, remain largely unknown. This study is aimed at evaluating the association between HEV seropositivity and consumption of self-grown foods in the United States.

**Methods:**

Cross-sectional data was extracted from the 2009–2012 National Health and Nutrition Examination Survey (NHANES). Data from the dietary interview and the serum HEV IgG and IgM enzyme immunoassay test results were linked and examined. Univariate and multivariable logistic regression models were used to evaluate the significance and effect size of an association between self-grown food consumption and hepatitis E seropositivity.

**Results:**

The estimated HEV seroprevalence in the civilian, noninstitutionalized US population was 6.6% in 2009–2012, which corresponds to an estimated hepatitis E national seroprevalence of 17,196,457 people. Overall, 10.9% of participants who ingested self-grown foods had positive HEV antibodies versus 6.1% of participants who did not consume self-grown foods (*P* < 0.001; odds ratio (OR) 1.87; 95% CI 1.41–2.48). In the age-stratified multivariable analysis, the correlation between ingesting self-grown foods and HEV seropositivity was significant for participants 40–59 years old, but not overall, or for those < 40 years or ≥60 years.

**Conclusions:**

Ingesting self-grown food, or simply the process of gardening/farming, may be a source of zoonotic HEV transmission.

## 1. Introduction

HEV is an enterically transmitted acute viral hepatitis and major cause of sporadic and epidemic hepatitis worldwide [1–3]. Infection by HEV is typically self-limited and resolves within 4–6 weeks; however, the severity of infection ranges from subclinical to fulminate hepatitis [2, 4, 5]. Hepatitis E is unique in its severity among pregnant women, who may face up to a 28% mortality rate, and the immunocompromised, who frequently progress to chronic hepatitis E without antiviral treatment [6–8]. Although identified clinical cases are rare, asymptomatic hepatitis E infection is now known to be widespread in industrialized nations [1, 4].

HEV transmission is linked to fecally contaminated drinking water [9, 10]. These cases are usually reported as part of a large-scale outbreak, often following a flood or another natural disaster in developing countries [5, 11]. The modes of HEV transmission in industrialized countries, including the United States, remain largely unknown. For years, it was suspected that HEV infections diagnosed in industrialized nations stemmed from travel to hyperendemic regions, especially in South Asia and North Africa [2]. Recent studies have demonstrated autochthonous (locally acquired) infection in several industrialized countries [12–14]. Unlike hepatitis A virus, HEV is not readily transmitted by person-to-person contact; therefore, it is postulated that these autochthonous infections originate from a zoonotic source [15].

Hepatitis E is recognized as a zoonotic disease [16]. Swine are a known HEV reservoir and many wild and domestic animals have been connected to transmission [5, 10, 16]. People whose occupations involve working with animals, especially livestock, have higher rates of HEV seropositivity [17, 18]. More recent studies support zoonotic transmission of HEV from domestic and pet animals [16]. Foodborne transmission has also been clearly described as a mode of sporadic zoonotic transmission in industrialized countries. Reports highlight consumption of raw or undercooked pork products (wild and domestic), game meats, shellfish, and produce as possible sources of human HEV infection [4, 10, 19–21]. We propose that humans may be exposed to HEV through self-grown foods, such as fruits and vegetables from a home garden contaminated by a zoonotic source. Our aim is to use cross-sectional data to evaluate the association between HEV seropositivity and consumption of self-grown foods.

## 2. Materials and Methods

Using data collected in the 2009–2012 National Health and Nutrition Examination Survey (NHANES), we performed a cross-sectional study to assess whether ingesting self-grown foods is associated with detection of HEV-specific IgM and IgG. The NHANES sample is a stratified multistage probability cluster designed to represent the total civilian noninstitutionalized US population. Probability sampling weights are applied to collected data to account for oversampling and nonparticipation and are used to calculate national estimates. Standardized interviewers, physical examinations, and tests of biologic samples are used to collect data in five major categories: demographics, dietary information, physical and dental examinations, laboratory tests, and questionnaires. Additional details concerning NHANES sampling and survey design can be found in the NHANES section of the Centers for Disease Control and Prevention (CDC) website [22].

The NHANES questionnaire asks participants to report demographics including age, gender, race/ethnicity, and birthplace. The race/ethnicity is reported in five categories: Mexican American, other Hispanic, non-Hispanic White, non-Hispanic Black, or other/multiracial. As for the dietary questionnaire, two dietary interviews were administered to all participants. The first interview was administered in person at the designated NHANES site. The second interview was administered over the phone 3–10 days later. Proxy interviews were administered for children 1–11 years old and for individuals unable to reliably answer questions themselves. During each dietary interview, participants were asked to report the types and sources of each food item eaten over the previous 24-hour period. Food items grown, caught, or hunted by the participant or someone the participant knew were categorized as self-grown foods. The recorded self-grown foods were then subcategorized into groups, which included fruits, vegetables, meat products, dairy, and grains.

HEV-specific IgM and IgG serologic tests were performed on all participants ≥ 6 years using enzyme immunoassay (EIA) kits provided by DSI—Diagnostics System Italy of Saronno, Italy. The CDC reported the IgM assay (DS-EIA-ANTI-HEV-M) as having 98% sensitivity and 95.2% specificity, and the manufacturer reported the IgG assay (DS-EIA-ANTI-HEV-G) as having 100% sensitivity and 97.5% specificity [13]. Detailed specimen collection and processing protocols can be found in the NHANES Laboratory Procedures Manual [23]. Further information concerning the anti-HEV EIA kits can be found in the CDC Laboratory Procedure Manuals for HEV testing [24, 25].

### 2.1. Statistical Methods

The raw number of survey participants is reported to describe the inclusion and exclusion of subjects from combining data from the demographic questionnaire, dietary interview, and laboratory NHANES datasets. The NHANES demographic and dietary questionnaires are available online [25–27]. All further analyses and seroprevalence estimates accounted for the NHANES survey design and included appropriate survey weights, which accounted for the differential probabilities of selection, nonresponse, and oversampling. Univariate analysis of the respective variables and seroprevalence was conducted first with standard errors estimated by the Taylor series linearization. Comparison of continuous variables was performed using Student's *t*-statistic. Univariate and multivariable logistic regression evaluated the significance and effect size of investigated independent variables with hepatitis E seropositivity (either IgG or IgM positive) as the dependent variable. The independent variables evaluated include: ingestion of self-grown food, sex, race/ethnicity, and foreign versus US born. The NHANES race/ethnicity categories were used with the modification that “Mexican–American” and “other Hispanic” categories were combined.

The seroprevalence by age was calculated and used to estimate a force of infection method as described by Shkedy et al. [28]. Rates of both self-grown food consumption and hepatitis E seropositivity were evaluated to identify whether self-grown food was significantly associated with hepatitis E seropositivity among various age groups. These age groups were based on the force of infection curve. An alpha level of 0.05 was utilized to determine statistical significance. Analyses were conducted using SAS 9.3 (SAS Institute, Cary, NC). The study was reviewed and deemed exempt by the responsible institutional review board because the data is deidentified and publicly available online.

## 3. Results

20,293 individuals participated in the 2009–2012 NHANES survey; 16,984 were ≥6 years old and therefore eligible to participate. Out of all eligible individuals, 14,951 had recorded HEV serologies. Participants with insufficient blood drawn and incomplete laboratory exams do not have recorded HEV serologies. Serum analysis showed that 894 (6.0%) individuals were positive for HEV-specific IgG and 146 (1.0%) were positive for HEV-specific IgM. Evaluating participants positive for either HEV-specific IgG or HEV-specific IgM yielded a seroprevalence of 6.6% (95% confidence interval (CI) 5.8%–7.4%), which corresponds to an estimated national HEV seroprevalence of 17,196,457 people (95% CI 14,561,770–19,831,144) in the civilian, noninstitutionalized US population.

**Figure 1:**
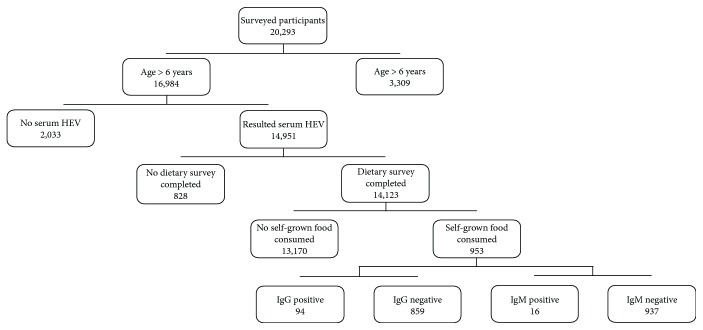
Participant analysis flow diagram, NHANES 2009–2012.

As for the nutritional component, 18,273 out of 20,293 survey participants completed the dietary interview. Approximately 7.3% (95% CI 6.2%–8.5%) of participants reported eating self-grown food, which represents an estimated 24,091,324 (95% CI 19,339,036–28,843,612) people nationally. Those who reported eating self-grown foods were older (median age 52.0; interquartile range (IQR) 36.5–66.0) than the remainder of the population (median 39.1; IQR 22.3–55.1; *P* < 0.001). All participants with both completed dietary interview and HEV serologies (14,123 out of 14,951) were included for further analysis (Figure 1).

The dietary interviews of participants with resulted HEV serologies included 317,416 foods. A total of 1833 items were reported in the self-grown food category. Self-grown foods reported by HEV-seropositive participants included fruits (45%), vegetables (23%), meat products (15%), dairy products (9%), honey/sweeteners (5%), and grains (3%).

Approximately 9.7% (standard error of percent (SEP) 1.2) of participants who ingested self-grown foods had detectable HEV-specific IgG compared to 5.4% (SEP 0.2) of participants who did not consume self-grown food (*P* < 0.001; OR 1.89; 95% CI 1.46–2.44). HEV-specific IgM was detected in 1.7% (SEP 0.52) of those who reported consuming self-grown food, versus 1.0% (SEP 0.2) in those who did not (*P* = 0.21; OR 1.66; 95% CI 0.76–3.66). When evaluating for any HEV antibodies (IgG or IgM), participants who reported eating self-grown foods had 10.9% seropositivity versus 6.1% in participants who did not consume self-grown foods (*P* < 0.001; OR 1.87; 95% CI 1.41–2.48). In the age-stratified multivariable analysis, however, the correlation between ingesting self-grown foods and HEV seropositivity was significant for participants 40–59 years old (OR 1.70; 95% CI 1.06–2.74), but not overall, or for those < 40 years or ≥60 years.

**Figure 2:**
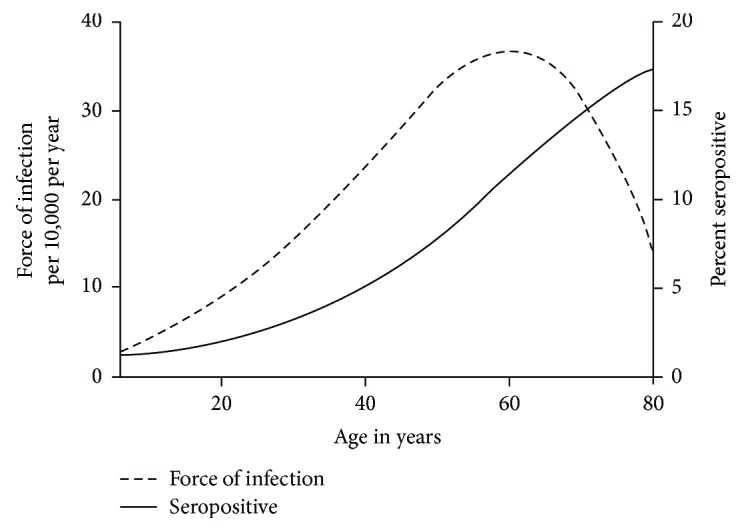
HEV-estimated force of infection and seropositivity by age for 2009–2012 NHANES participants.

In addition to consuming self-grown foods, several other factors were found to be associated with HEV seropositivity. HEV-seropositive individuals were found to be older, with a median age of 57.6 years (IQR 45.0–69.0), compared to a median age of 40.7 years (IQR 23.7–56.3; *P* < 0.001) in the overall serum-tested population. This finding is consistent with previous reports [29, 30]. Furthermore, the estimated seropositivity steadily increases throughout life, with a maximum force of infection peaking at 36 per 100,000 in the 6th decade of life. The prevalence and force of infection by age are presented in Figure 2.

Univariate and multivariable-adjusted odds ratios for HEV seropositivity by selected characteristics are shown in Table 1, with multivariable-adjusted odds ratios further stratified by age in Table 2. Birth country was a significant associative factor, as the seroprevalence of participants born outside of the Unites States (9.3%; SEP 0.88; OR 1.78; 95% CI 1.23–2.58) was higher than that of US-born participants (6.0%; SEP 0.45). In gender comparisons, young females (6–39 years) were found to have higher HEV seropositivity compared to males of the same age group (OR 1.44, 95% CI 1.03–2.02). Finally, non-Hispanic Black participants had lower rates of HEV infection than White participants (OR 0.60, 95% CI 0.46–0.79). Other race/ethnicity comparisons were not statistically significant.

## 4. Discussion

This study demonstrates an association between ingesting self-grown food and seropositivity for HEV. Based on the 2009–2012 National Health and Nutrition Survey (NHANES), the estimated HEV seroprevalence of the US civilian and noninstitutionalized population is 17.2 million people (6.6%). Since the best-characterized modes of HEV transmission—drinking fecally contaminated water and working with infected swine—are relatively rare in the US, exploring other modes of transmission is critical to understanding HEV epidemiology. HEV transmission through contaminated self-grown produce is plausible based on the ever-expanding range of identified zoonotic hosts and identification of HEV on agricultural products [19, 20, 31].

A mounting body of evidence has demonstrated that other animal species play a major role in HEV transmission to humans [10, 16, 32]. Despite documentation of cross-species HEV transmission to humans, the mechanisms of infection remain unclear [33]. Swine are a well-known reservoir, as multiple studies have reported positive HEV serologies in swine worldwide [5, 34]. Recent reports have expanded the range of possible zoonotic hosts after finding similar strains of HEV in cattle, deer, rabbits, wild boar, birds, rats, and several other animals [16, 33].

As the list of identified zoonotic sources has expanded, so have the possible modes of transmission. Consuming raw or uncooked meats has been linked to cases of human HEV in multiple countries [4, 10, 21, 35]. Occupations that require frequent contact with livestock are known to increase the likelihood of HEV infection [17, 18, 36, 37]. Gardeners who work with, or live near, these animals may be spreading HEV through irrigation runoffs, or transferring the virus directly from animal feces to garden products. Therefore, even the process of gardening (planting, weeding, and preparing soil) may be a cause of HEV exposure.

Recent North American and European studies have demonstrated that HEV can be found on agricultural products such as raspberries, strawberries, lettuce, and other vegetables at the primary production level and point of sale [19, 20, 31]. Teshale and Hu reported an increased HEV prevalence in Americans who consumed more green leafy vegetables or lettuce salad [3]. In our study, fruits and vegetables comprised two-thirds of all reported self-grown foods, with tomatoes, cucumbers, and apples representing the most commonly reported self-grown food items among seropositive participants. Still, it is important to bear in mind that the NHANES nutritional survey is a brief snapshot of participant-reported foods ingested by individuals. The behavior of gardening, farming, and ingesting self-grown foods is likely a pattern that persists across all seasons and types of produce.

In our univariate analysis, consuming self-grown food was found to be significantly associated with HEV seropositivity in all age ranges. The multivariable logistic model, however, found ingesting self-grown food to be significantly associated with positive HEV serologies only for participants aged 40–59 years (not participants < 40 years or ≥60 years). This finding is consistent with the force of infection, which peaks within the same age range. These results suggest that people aged 40–59 years may be at increased risk for acquiring HEV infection, although the reason for this remains unclear.

This study identified other factors associated with HEV seropositivity. As expected, increasing age corresponded with increased HEV seroprevalence. Gender played a role in HEV infection in the younger population (6–39 years), as females were found to have higher HEV seroprevalence than males. Among all races/ethnic groups, our results, in concordance with other reports [12, 29], found the highest rates of HEV seroprevalence in the non-Hispanic White population. Finally, foreign birth was significantly associated with positive HEV serologies only for participants in the youngest age category (6–39 years). This finding is consistent with the epidemiology of HEV infection in developing nations, where young adults have the highest rates of HEV detection and disease [38–40]. Unfortunately, travel history was not included in the NHANES survey and we are therefore unable to determine whether patients have travelled outside of the United States to endemic regions.

This study is one of few nationally representative studies looking at HEV transmission in the United States. Furthermore, NHANES' standardized sampling methods, questionnaires, and protocols make it a reliable and powerful source for national epidemiological studies. It is important, however, to interpret our findings in the context of their limitations. First, this is a cross-sectional study and therefore cannot demonstrate self-grown food consumption as a cause of HEV infection. Second, the data may be subject to recall bias, as participants were asked to remember foods eaten during two 24-hour periods. Third, past studies have detected HEV-specific IgG in the blood up to 14 years after infection [5, 41], which helps explain why HEV seroprevalence rises throughout life, but may also mask associations, as there could be a lag between exposure to HEV and detection of HEV-specific antibodies. Finally, it is possible that there are other confounding factors that should be considered, such as geography (i.e., urban versus rural region), occupation, and genotype data that are not reported by NHANES. These confounding variables could be useful in describing HEV epidemiology and associated factors. Documenting the distribution of HEV genotypes could be particularly helpful in clarifying the proportions of travel-related and autochthonous HEV infections in the United States.

In conclusion, hepatitis E virus is prevalent in the United States, with an estimated national seroprevalence of 6.6%. Middle-aged participants (40–59 years) in the 2009–2012 NHANES dietary survey who reported consuming self-grown foods, such as vegetables from a home garden, were more likely to be seropositive for HEV. Self-grown foods exposed to HEV-contaminated animal feces may be a source of zoonotic transmission to humans in industrialized countries. In addition, the process of growing produce and working in HEV-contaminated soil may be a mode of zoonotic HEV transmission to humans. As with all foods, self-grown foods should be carefully cleaned before consumption, especially for people at high risk for HEV-related complications, such as pregnant women and immunosuppressed patients. Further investigation concerning HEV transmission is an essential step toward developing effective preventive measures against hepatitis E infection.

## Figures and Tables

**Table 1:** Odds ratios and adjusted odds ratios with 95 percent confidence intervals (CI) for HEV seropositivity by selected characteristics, NHANES 2009–2012.

	Odds ratio (95% CI)	Adjusted odds ratio (95% CI)
Self-grown food	1.87 (1.41–2.48)	1.32 (0.96–1.81)
Foreign born	1.61 (1.24–2.10)	1.78 (1.23–2.58)
Female versus male	1.13 (0.96–1.34)	1.08 (0.91–1.29)
Age	1.50 (1.44–1.57)	1.50 (1.43–1.57)
Race/ethnicity		
Non-Hispanic Black versus White	0.48 (0.38–0.61)	0.60 (0.46–0.79)
Hispanic versus White	0.81 (0.63–1.04)	0.91 (0.65–1.27)
Other versus White	1.21 (0.93–1.58)	1.21 (0.86–1.70)

**Table 2:** Adjusted odds ratios with 95 percent confidence intervals (CI) for HEV seropositivity using age-stratified multivariable logistic regression, NHANES 2009–2012.

	Age 6–39 years	Age 40–59 years	Age ≥ 60 years
Self-grown food	1.43 (0.50–4.10)	1.70 (1.06–2.74)	1.15 (0.79–1.67)
Foreign born	4.46 (3.09–6.44)	1.14 (0.62–2.08)	1.49 (0.96–2.33)
Female versus male	1.44 (1.03–2.02)	0.94 (0.65–1.36)	1.07 (0.82–1.41)
Race/ethnicity			
Non-Hispanic Black versus White	0.49 (0.28–0.83)	0.83 (0.54–1.28)	0.50 (0.33–0.76)
Hispanic versus White	0.74 (0.44–1.23)	1.35 (0.84–2.17)	0.67 (0.43–1.04)
Other versus White	1.23 (0.69–2.17)	1.74 (0.92–3.31)	0.80 (0.43–1.47)

## Data Availability

Hepatitis E serologies and dietary surveys analyzed for this article are available from the Centers for Disease Control and Prevention's National Health and Nutrition Examination Survey website (https://wwwn.cdc.gov/nchs/nhanes/search/nnyfs12.aspx).
